# High Crimean-Congo hemorrhagic fever incidence linked to greater genetic diversity and differentiation in *Hyalomma marginatum* populations in Türkiye

**DOI:** 10.1186/s13071-024-06530-z

**Published:** 2024-11-19

**Authors:** Olcay Hekimoğlu, İsmail K. Sağlam

**Affiliations:** 1 Faculty of Science, Department of Biology, Division of Ecology, Hacettepe University, 06800 Beytepe, Ankara Türkiye; 2 Faculty of Science, Department of Molecular Biology and Genetics, Koc University, 34450 Ýstanbul, Türkiye

**Keywords:** *Hyalomma marginatum*, Türkiye, CCHF, RAD-Seq

## Abstract

**Background:**

Ticks are crucial vectors of a wide range of pathogens, posing significant threats to human and animal health globally. Understanding the genetic basis of tick biology and host–parasite interactions is essential for developing effective control programs. This study investigates the fine-scale genetic structure of *Hyalomma marginatum* Koch, 1844, the primary vector of Crimean-Congo hemorrhagic fever (CCHF) in Türkiye. Despite its significant public health importance, information regarding its population structure and genetic diversity is quite limited.

**Methods:**

We used restriction site-associated DNA sequencing (RAD-Seq) to obtain genome-wide sequence data from 10 tick populations in Türkiye, collected from regions with low, moderate, and high incidence rates of CCHF. Based on these data, we determined population structure and diversity of populations using principal component analysis (PCA) and admixture analysis. Furthermore, we calculated pairwise F_ST_ and utilized discriminant analysis of principal components (DAPC) to understand genetic differentiation between populations.

**Results:**

PCA and admixture analysis indicated minimal genetic structure between populations, but we detected notable genetic differentiation and high genetic diversity from regions with high CCHF rates. Furthermore, our DAPC identified 31 significant single-nucleotide polymorphisms (SNPs) associated with regions with high CCHF incidence, with 25 SNPs located near genes involved in critical biological functions such as nucleic acid binding, transmembrane transport, and proteolysis. These findings suggest that genetic variations in these regions may confer adaptive advantages in environments with high pathogen loads.

**Conclusions:**

This study provides the first comprehensive analysis of *H. marginatum* genetic diversity in Türkiye, revealing significant differentiation in populations from CCHF-endemic regions. These results underscore the importance of considering fine-scale genetic diversity to fully understand the drivers of genetic variation in ticks and their implications for vectorial capacity.

**Graphical Abstract:**

**Figure:**
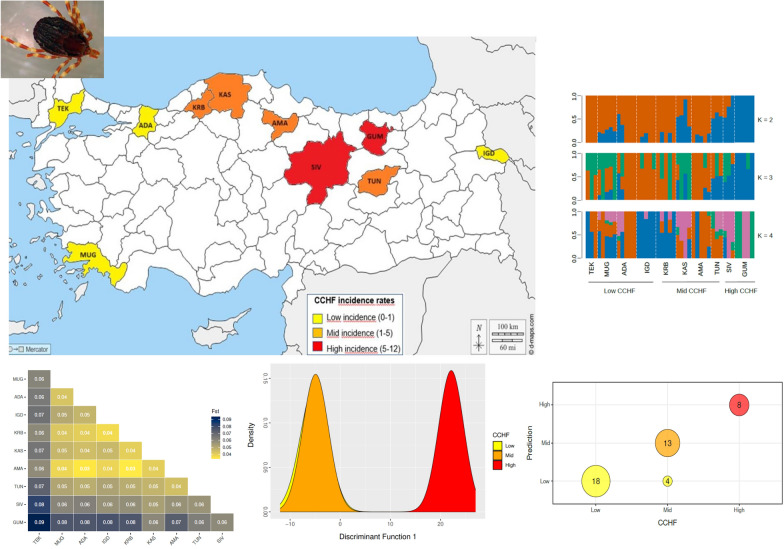

**Supplementary Information:**

The online version contains supplementary material available at 10.1186/s13071-024-06530-z.

## Background

Ticks play a crucial role as vectors of human and animal pathogens, transmitting a greater variety of infectious agents than other blood-feeding arthropods. Despite the significant global threat of tick-borne diseases, our understanding of the basic biology of many tick species and the genetic basis of host–parasite-–tick interactions remains limited, hindering the development and implementation of effective tick control programs. Over the past 20 years, it has been emphasized that new approaches in vector biology studies are necessary and that genetic resources must be enhanced in various tick species [1, 2]. For example, as a result of the sequencing of the first tick genome, that of *Ixodes scapularis*, efforts to control ticks and the diseases they transmit have expanded rapidly [3].

Population genetics studies can provide insight into the evolutionary dynamics of species, and therefore can be important for understanding the spatial dynamics of pathogen specificity, host adaptation, and resistance to chemical acaricides in tick species [4]. Additionally, local adaptation observed in vector and pathogen populations is a significant factor influencing epidemiology. Research on the vector competence of different arthropod species has revealed significant geographical variations in their capacity to transmit arboviral diseases [4–8]. Furthermore, geographical variation has also been reported as an important mechanism for acquiring, maintaining, and transmitting pathogens in different tick species [3, 9, 10].

*Hyalomma marginatum* Koch, 1844, is a tick species widely distributed in the Palearctic region and is the vector of Crimean-Congo hemorrhagic fever (CCHF) [11]. In Türkiye, the vectorial importance of *H. marginatum* increased with the first CCHF case reported in 2002. According to data from the Turkish Ministry of Health, 10,562 cases were recorded between 2008 and 2017, resulting in 501 deaths (https://hsgm.saglik.gov.tr/tr/dokumanlar-zoonotik.html). Despite CCHF being the most widely distributed tick-borne viral disease in the world [12], studies on the genetic structure of its vector are limited. Previous studies conducted by our group using microsatellite markers revealed limited genetic diversity among populations [13, 14], restricting our ability to understand fine-scale patterns of genetic structure and diversity of this species across Türkiye.

The main objective of this study was to investigate the fine-scale genetic structure of *H. marginatum* populations across Türkiye and to understand whether spatial patterns of genetic diversity could provide insight into vectorial capacity and the observed differences in CCHF incidence rates across the country. To this end, we generated genome-wide sequence data using restriction site-Associated DNA sequencing (RAD-Seq) [15–17] and compared patterns of genetic diversity and differentiation between populations and tested for unique polymorphisms associated with increased incidence of CCHF.

## Methods

### Sampling, morphological and molecular identification

**Fig. 1:**
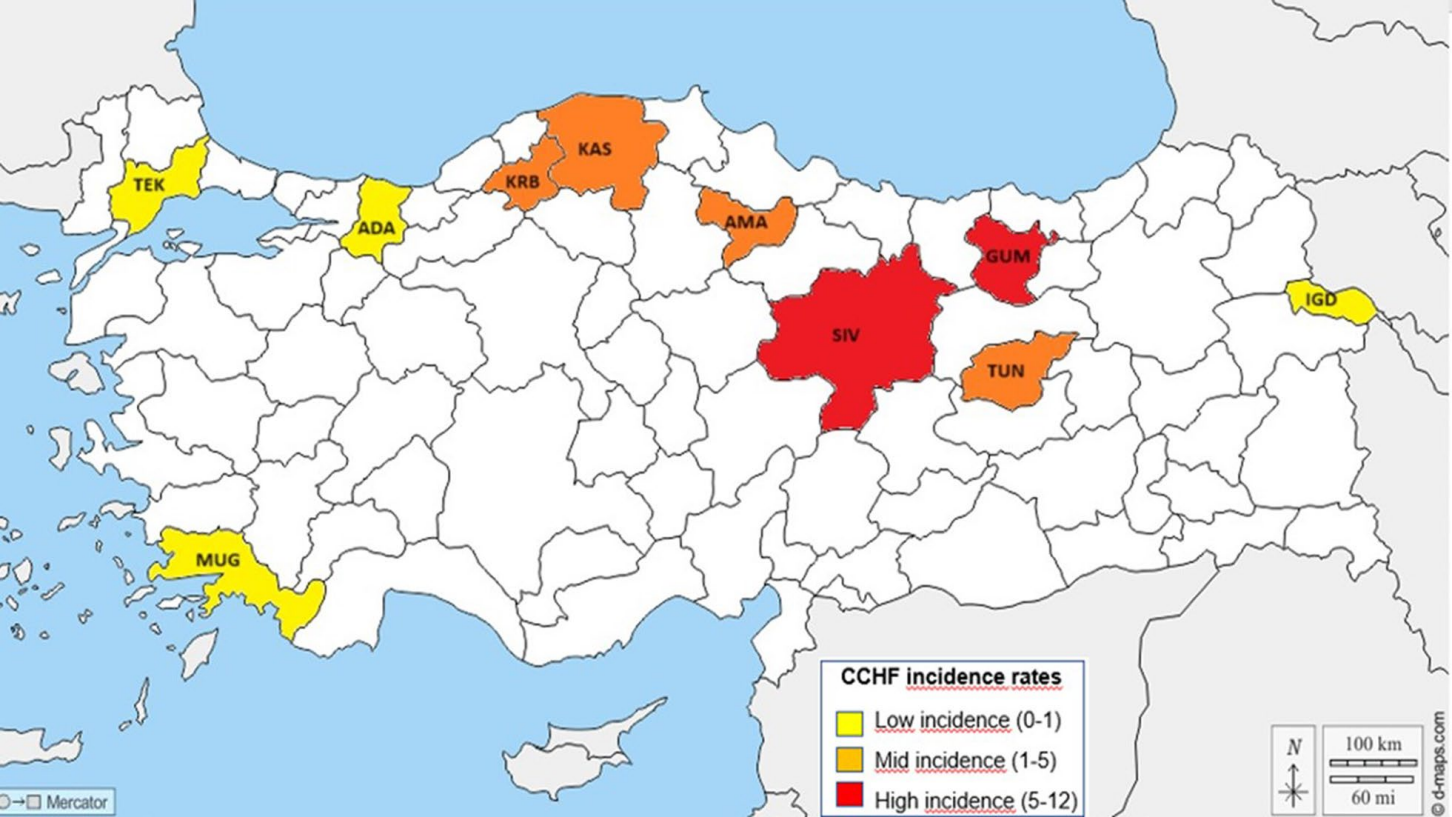
Map of the collection localities, with colors representing the incidence rate of confirmed CCHF cases from 2002 to 2017, according to reports from the Turkish Ministry of Health (https://hsgm.saglik.gov.tr/tr/dokumanlar-zoonotik.html). Abbreviations of the provinces: *ADA* Adapazarý, *AMA* Amasya, *GUM* Gümüºhane, *IGD* Iðdýr, *KAS* Kastamonu, *KRB* Karabük, *MUG* Muðla, *SIV* Sivas, *TEK* Tekirdað, *TUN* Tunceli

Tick samples were collected from 10 locations across Türkiye between 2018 and 2023 (Fig. 1; Supplementary Table 1). These locations covered the entire known distributional range of the species in Türkiye, including areas both endemic and non-endemic to CCHF according to incidence maps from the Turkish Ministry of Health (https://hsgm.saglik.gov.tr/tr/dokumanlar-zoonotik.html). Although *H. marginatum* has been reported to be present in all geographical regions of Türkiye [18, 19], recent studies utilizing molecular approaches to identify species within the genus have indicated that it is mainly distributed in the central, northern, and eastern regions of the country [20–25]. This tick species has also been reported in the western (Aegean) and Thrace regions of Türkiye, where CCHF incidence rates are relatively low [18, 24, 26–28]. The selected regions in this study included areas with low incidence rates (0–1: Adapazarý, Iðdýr, Muðla, and Tekirdað) moderate incidence rates (mid-CCHF; 1–5: Kastamonu, Karabük, Amasya, and Tunceli), and high incidence rates (5–12: Gümüºhane and Sivas).

Morphological identification was carried out using identification keys [11, 29, 30]. To verify species delimitation based on morphological identification and to resolve any potential ambiguities, we double-checked species status using molecular identification. Molecular identification of individuals was conducted using 16S ribosomal DNA (rDNA) markers following the primers and protocols of Mangold et al. [31]. DNA extraction was performed using the GeneJET Genomic DNA Purification Kit (Thermo Fisher Scientific), and DNA concentration was measured with a Qubit 4 Fluorometer and Qubit dsDNA HS Assay Kit (Invitrogen). The 16S polymerase chain reaction (PCR) products were sent to Macrogen for sequencing, and the resulting sequences were inspected and edited using Sequencher v5.4.6 (Gene Codes Corporation, http://www.genecodes.com).

### RAD-Seq and alignment

Ninety-five DNA samples were chosen for RAD-Seq based on their DNA quality. RAD libraries were prepared using the PstI restriction enzyme and sequenced at 10× on the Illumina platform. Library preparation and sequencing were carried out by Floragenex (Eugene, OR, USA). Sequences were then sorted into individuals by matching forward and reverse fastq files utilizing unique 10-base-pair (bp) barcodes. Sequences were mapped to the *Hyalomma anatolicum* [32] reference genome using the BWA-MEM algorithm [33]. Samtools v1.14 [34] was employed to convert the outputted SAM (Sequence Alignment Map) files into BAM (Binary Alignment Map) files, and subsequently to sort and index the aligned reads. Sequencing duplicates were marked using the MarkDuplicates module of Picard v2.22.1.

### Single-nucleotide polymorphism (SNP) discovery and genotyping

Variant discovery and genotyping were carried out using the ANGSD (Analysis of Next Generation Sequencing Data) probabilistic framework [35]. We calculated genotype likelihoods (-GL 1), genotype probabilities (-doGlf 2), and minor allele frequencies (-doMaf 1) in ANGSD using a minimal quality score of 20 (-minQ 20) and mapping quality of 10 (-minMapQ 10). Polymorphic sites were calculated based on a minor allele frequency threshold of 0.05 and a significance threshold of 1 × 10^−6^ (-minMAF 0.05; -SNP_pval 1e–06). The minimum read depth was set to an average of 6× per population, and sites not present in at least 50% of individuals in each population were filtered out. Identical parameters were used to call genotypes in VCF (Variant Call Format) (-doGeno 4 -doVcf 1), employing a population prior (-doPost 1) and a posterior probability cutoff of 95% (-postCutoff 0.95).

### Genetic diversity

Genetic diversity and neutrality of populations were measured by calculating theta Pi (the average number of pairwise nucleotide differences .p) and Tajima’s D statistics in ANGSD in 50-kilobase (kb) overlapping windows, with a step size of 25 kb. To calculate these statistics, we first estimated a folded-SFS (site frequency spectrum) for each population (doSAF) and used this as a prior to determine theta and Tajima’s D values (saf2theta). The .p values were converted into per-site values by dividing each statistic by the number of sites in the window. Genome-wide values of .p and Tajima’s D were obtained for each population by averaging across all windows.

### Genetic structure

Genetic structure among populations was investigated through principal component analysis (PCA) based on the genetic covariance matrix between individuals calculated in PCAngsd [36] using the genotype likelihood values calculated in ANGSD. Principal component axes summarizing genetic structure were then derived from this matrix using classical eigenvalue decomposition in R version 4.2.0 [37] and visualized using ggplot [38]. In order to assess shared ancestry among populations, we performed admixture analyses for different values of K (2–10) using NgsAdmix [39] based on genotype likelihoods. We executed NgsAdmix 10 times per K value, and used the likelihood values from each run to determine the most likely K, following the method outlined in Evanno et al. [40].

### Genetic differentiation

Genetic differentiation among populations was assessed using the global Fixation Index (F_ST_) [41, 42], which considers information from all loci equally. To compute pairwise F_ST_ values between populations, we calculated the site frequency spectrum of each population in ANGSD (doSAF) and then used the RealSFS module [35] to determine the two-dimensional SFS and calculate pairwise weighted global F_ST_ values based on 50-kb overlapping windows, with a step size of 25 kb.

To determine whether patterns of genetic differentiation matched a pattern of isolation by distance, we conducted a Mantel test between geographical and genetic distances. The linearized pairwise F_ST_ matrix [F_ST_/(1- F_ST_)] served as the response variable, and the pairwise Euclidean distances between populations were the predictor variable. All calculations for the Mantel tests were performed in R using the adegenet package [43].

To determine genetic variation between populations that could be contributing to differences in CCHF rates, we used discriminant analysis of principal components (DAPC) to determine the primary axis of variation among individuals classified according to CCHF rates (low, mid, high). We utilized the adegenet package in R [43] to perform DAPC, with CCHF rates provided as an initial grouping variable. To assess DAPC's effectiveness in categorizing individuals based on CCHF rates, we visualized the first discriminant function and analyzed the contributions (loadings) of each site to differentiation in CCHF rates. SNPs with loadings above the 99% quantile of the null distribution were marked as significant, and for each significant SNP, we determined the closest accessions within a 100-kb window based on the *H. anatolicum* reference genome [32]. Exome sequences of the discovered accessions were then blasted to the National Center for Biotechnology Information (NCBI) database, restricting the search to order Ixodidae, and only hits with 85% coverage and 90% identity to the query sequence were regarded as successful and annotated. Lastly, we cross-referenced successful annotations with VectorBase (https://vectorbase.org/) to determine the functional properties of determined genes.

## Results

Sequencing yielded a total of 807,624,459 raw reads, averaging 8,501,310 raw reads per sample. Individual alignment statistics are presented in Supplementary Table 2. Based on alignment statistics, we excluded individuals with an alignment success rate of less than 85% or with fewer than 100,000 aligned reads. (Supplementary Table 2). From the remaining individuals, we randomly selected three to five individuals from each population to standardize the sample size per population. The final dataset consisted of 43 individuals representing 10 populations. Across all populations, we identified 34,314 high-probability (*P* < 10^−12^) SNPs with a minor allele frequency over 0.05.

### Genetic diversity

**Fig. 2:**
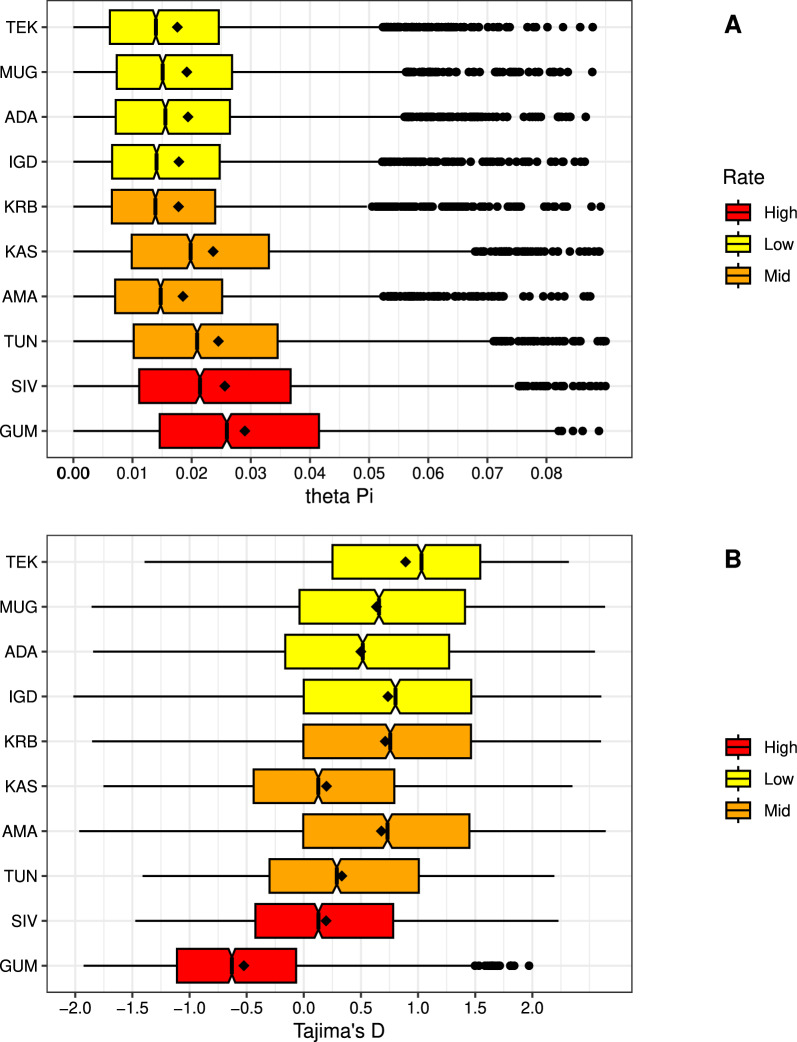
Genetic diversity analyses of 10 *H. marginatum* populations across Türkiye. **A** Theta pi (nucleotide diversity), **B** Tajima’s D

Patterns of genetic diversity were consistent across all populations and chromosomes (Supplementary Fig. 1). Genome-wide average èð values were generally high, ranging from 0.018 to 0.033 (Fig. 2A, Supplementary Table 3). The highest values were observed in regions with the highest CCHF incidence rates (Gümüºhane [GUM] and Sivas [SIV], Fig. 2A). Genome-wide mean values of Tajima's D were positive for all populations except GUM, ranging from 0.19 to 0.90, and showed minimal deviation from neutrality. In contrast, the GUM population had a negative Tajima's D value of –0.54, but it also did not deviate significantly from neutrality (Fig. 2B, Supplementary Table 4).

### Genetic structure

**Fig. 3:**
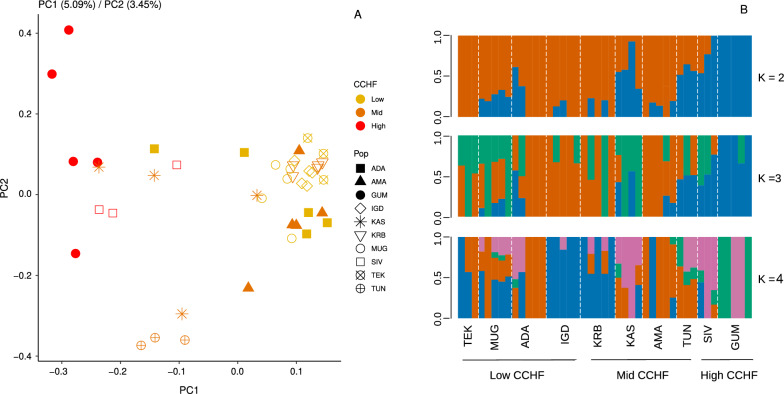
Genetic structure analyses of 10 *H. marginatum* populations across Türkiye. **A** Principal component analysis (PCA) displaying the clustering of 10 populations, colored according to CCHF incidence rates. **B** Estimated proportions of admixture for K = 2–4

Our PCA analysis showed only a loose structure between *H. marginatum* populations across Türkiye, as genetic variation was mostly scattered across the first two principal component axes. However, populations from high CCHF areas (GUM and SIV) were the most divergent and separated from other populations across PC1 (Fig. 3A). Admixture results also revealed minimal structure between populations supporting a K value of 2 and showing high levels of shared ancestry across populations (Fig. 3B). Similar to PCA, GUM and SIV populations were the most divergent, with GUM and TEK being the only population with mono-ancestry based on K = 2 (Fig. 3B).

### Genetic differentiation

**Fig. 4:**
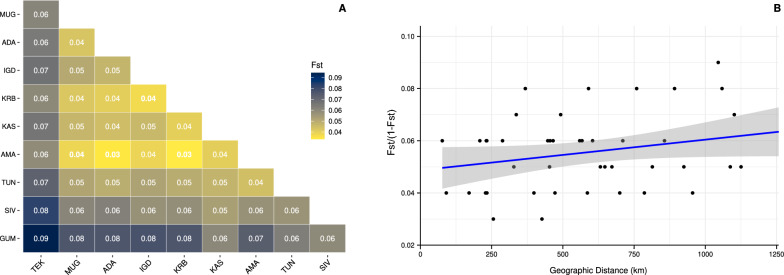
Genetic differentiation analyses of 10 *H. marginatum* populations across Türkiye. **A** Pairwise F_ST_ comparisons between populations. The legend on the right-hand side indicates the F_ST_ value-based coloring on a scale from 0.04 (yellow) to 0.09 (dark blue), where 0.04 represents the lowest differentiation and 0.09 represents the highest differentiation. **B** Isolation by distance (IBD). The genetic distance of F_ST_ versus geographical distance (km) is plotted for all pairwise comparisons among populations

Comparisons of pairwise F_ST_ values between populations indicated low genetic differentiation across Türkiye, as F_ST_ values mostly remained below 0.05 (Fig. 4A), and our Mantel test failed to capture any significant pattern of isolation by distance (*r* = 0.3113; *P* = 0.09) (Fig. 4B). The only notable exception was the GUM and TEK populations, which showed higher genetic differentiation from the other populations (F_ST_ = 0.06–0.09, Fig. 4A).

**Fig. 5:**
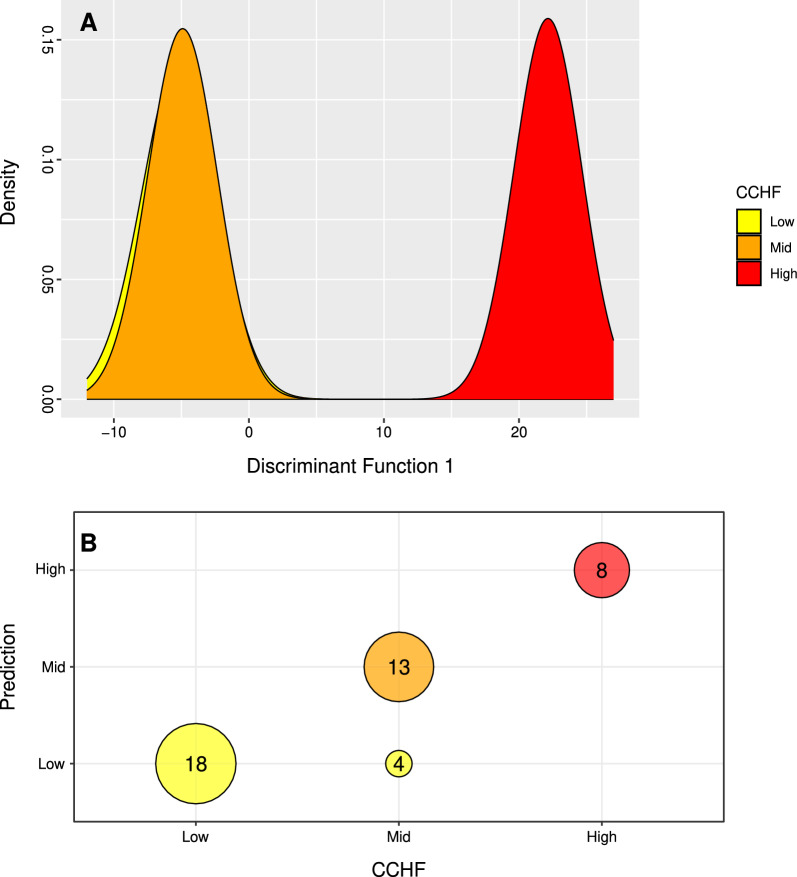
Discriminant analysis of principal components (DAPC) and posterior classification according to CCHF incidence rates. **A** DAPC plot. **B** The posterior classification of each sample in the three assigned groups

DAPC conducted based on prior CCHF groupings separated samples largely into two groups across the first discriminant axes with a high posterior probability of assignment (0.91, Fig. 5A). DAPC could not fully differentiate between samples within low- and mid-CCHF regions, as 24% of samples within the mid-CCHF regions were classified as low (Fig. 5B). Counter to this, samples within high-CCHF regions were highly distinct, with an assignment probability of 1 (Fig. 5A, B). Across DF1 we detected 31 SNPs significantly associated with this differentiation (Supplementary Fig. 2). Among the 31 SNPs, 25 were found within a 50-kb distance of 20 genes (Table 1).

**Table 1:** Details of site and function for the 25 SNPs involved in genetic differentiation

Chr	Pos	Distance (kb)	Gene accession	Annotation	Function
Chr1	546678	16.1	GWHTCBGJ000008	NA	NA
Chr1	1667546	18.9	GWHTCBGJ000021	NA	NA
Chr1	78328397	3.8	GWHTCBGJ001003	LOC119378975	NA
Chr1	88998120	5.7	GWHTCBGJ001135	LOC119181414	Nucleic acid binding, RNA binding
Chr1	118341223	1.4	GWHTCBGJ001437	NA	NA
Chr1	162837207	32.4	GWHTCBGJ002064	LOC119391178	NA
Chr1	184682818	7.4	GWHTCBGJ002310	LOC119384467	NA
Chr1	189636526	17.6	GWHTCBGJ002390	NA	NA
Chr1	211224298	5.7	GWHTCBGJ002643	LOC119403229	Nucleic acid binding, RNA binding
Chr1	232098945	32.9	GWHTCBGJ002884	LOC119455501	Nucleic acid binding; RNA–DNA hybrid ribonuclease activity; sodium:proton antiporter activity
Chr1	262319558	15.4	GWHTCBGJ003212	LOC119402299	NA
Chr1	267038345	4.2	GWHTCBGJ003249	LOC119466590	NA
Chr1	312641130	7.4	GWHTCBGJ003676	LOC125941854	Protein binding
Chr1	405995534	2.1	GWHTCBGJ004711	LOC119405712	Metallocarboxypeptidase activity; zinc ion binding
Chr1	465423344	16.2	GWHTCBGJ005280	LOC119160723	Nucleotide binding; aminoacyl-transfer RNA (tRNA) editing activity; valine-tRNA ligase activity; adenosine triphosphate (ATP) binding; ligase activity
Chr2	107494064	6.1	GWHTCBGJ00689	LOC119385606	Glycosyltransferase activity
Chr2	137404629	0.5	GWHTCBGJ007299	LOC135913654	NA
Chr2	205881586	3.4	GWHTCBGJ008473	LOC119386295	Actin binding
Chr3	36906022	1.1	GWHTCBGJ009205	LOC119381508	Autophagy
Chr3	175119797	2.6	GWHTCBGJ010989	LOC119384060	Mismatched DNA binding; ATP-dependent DNA damage sensor activity
Chr4	21293793	9.1	GWHTCBGJ011219	NA	NA
Chr4	25626549	2.8	GWHTCBGJ011244	LOC119168526	Regulation of posttranscriptional gene silencing
Chr5	71666402	2.6	GWHTCBGJ014276	LOC119394152	Signal transduction
Chr5	111180900	0.9	GWHTCBGJ014879	LOC119393542	Ferrochelatase activity
Chr7	28622543	7.6	GWHTCBGJ017900	LOC119391735	NA

## Discussion

In this study, we aimed to investigate the genetic differentiation and diversity of *H. marginatum* populations between CCHF-endemic and non-endemic regions of Türkiye. Our results revealed that CCHF-endemic regions have the highest level of genetic diversity among all sampling locations. F_ST_ and admixture analysis confirmed this, with the GUM population being the most genetically different population. This finding holds significance for two reasons: (1) Despite Türkiye's relatively small size and short distances between sampling localities, detecting distinct patterns of genetic variation is noteworthy. (2) Given that GUM and SIV have the highest CCHF incidence rates in the country, understanding the genetic differences in these regions is crucial for assessing their vectorial competence.

Previous studies on *H. marginatum* using microsatellite markers [13, 14] suggested low levels of genetic differentiation among populations, with no differences observed among localities according to CCHF incidence rates, and with TEK identified as the most genetically different population. In contrast, our study, using RAD-Seq, identified not only TEK but also GUM, the region with the highest CCHF incidence in Türkiye, as the most genetically diverse population. This discrepancy between the two studies may be attributed to the markers utilized. Although microsatellites are valuable for delineating genetically distinct populations, RAD-Seq is a more powerful tool in population genetics studies, allowing for more robust comparisons using significantly more loci.

Ticks play a crucial role in the transmission of various pathogens, and understanding their genetic variation is essential for determining vectorial capacity [44]. While comprehensive genome-wide studies on *H. marginatum* are limited, research on other tick species, such as *I. scapularis*, has offered valuable insights [45, 46]. For instance, RAD-Seq analyses revealed moderate genetic differences between reference colonies and natural populations of *I. scapularis*, with notable variation observed between regions with high and low Lyme disease incidence in the USA [3]. Interestingly, the highest genetic variability was detected in southern regions, where Lyme disease is non-endemic [47]. In contrast, our study identified the highest genetic variability in CCHF-endemic regions, indicating that these areas warrant further attention. Both our findings and those of Frederick et al. [47] were based on limited sampling across the genome. This underscores the need for more comprehensive genome-wide studies to accurately identify the genomic regions responsible for the observed patterns of variation.

Geographical variation significantly influences the genetic structure of ticks and is crucial for assessing disease transmission risks, developing effective control measures, and gaining insights into the evolutionary dynamics of vector species. Our study found no strong correlation between genetic diversity and geographical distance, suggesting that factors other than isolation by distance, such as host movements and environmental pressures, may play a significant role in shaping genetic diversity. While isolation by distance is a common explanation for geographical variability, it may not fully account for observed patterns in most tick species. Several studies have indicated that the relationship between genetic divergence and geographical distance is complex. For instance, Frederick et al. [47] found no strong correlation between genetic and geographical distance in *I. scapularis* populations, whereas Schoville et al. [48] observed a significant trend of isolation by distance in the genomic variation of *I. scapularis*, indicating increased genetic divergence over several hundred kilometers. In *Ixodes uriae* populations, genetic variation was detected within breeding cliffs, suggesting that tick dispersal depends more on host movements than local distances [49]. Similarly, Hekimoglu et al. [14] found no correlation between isolation by distance and genetic variation in *H. marginatum*.

In addition to geographical variation, hybridization and introgression are also known to affect patterns of genetic variation in tick species. Genetic exchange between different tick species has been well documented, particularly among *Ixodes* [50–52] and *Dermacentor* [53] species. Similarly, it has been suggested that hybrid forms can arise from mating between *Hyalomma* species, with these hybrids being transported to different regions by migratory birds [54]. Given the sympatric occurrence of *H. marginatum* with *Hyalomma excavatum*, *H. anatolicum*, and *H. asiaticum* in Türkiye, hybridization could be a significant factor explaining the genetic variability between *H. marginatum* populations observed in this study. Although this study did not cover hybridization between *Hyalomma* species, it is crucial to explore this topic further, as genetic mixing can facilitate or hinder the transfer of traits related to pathogen transmission and adaptation to different environments.

Another important factor that may contribute to genetic differentiation among tick populations is host specialization. This phenomenon has been demonstrated in other tick species, such as *I. scapularis*, which has led to varying incidence rates of Lyme disease [55, 56]. However, in our study, it is difficult to suggest this, as all samples were collected from the same host species (cow). Additionally, *H. marginatum* is a generalist tick species, which enables it to utilize a wide range of host species [11, 57]. Given Türkiye's geographical position as a migratory route for birds, along with the frequent movement of livestock due to animal husbandry or religious practices, high gene flow between populations can be expected, but we did not observe this in our study.

RAD-Seq has significantly improved our ability to detect genetic variation in vector populations and signals of selection [58, 59]. For instance, Frederick et al. [47] identified 10 variable loci in *I. scapularis* populations in the USA, with some loci located within annotated genes in the reference genome [47]. These loci potentially influence traits crucial for the tick’s vectorial capacity, such as pathogen transmission and host preference. Similarly, here we uncovered 31 candidate loci with higher tick population diversity in regions with high CCHF incidence in Türkiye. Twenty-five of these loci were within 50 kb of genes involved in various biological functions such as nucleic acid binding, transmembrane transport, and proteolysis (Table 1). The proximity of these loci to genes with diverse biological functions indicates that these genetic variations could confer adaptive advantages in the harsh environments where CCHF is prevalent. For example, enhanced autophagy and mismatch repair mechanisms could help ticks survive in environments with high pathogen loads [60, 61], while variations in genes involved in signal transduction and protein binding could affect their interactions with hosts and pathogens [44, 62]. The identification of loci associated with critical biological functions provides further insights into the genetic basis of vectorial capacity and adaptation. Several pathways in the tick genome, including the Toll, immunodeficiency (IMD), and Janus kinase/signal transducers and activators of transcription (JAK-STAT) immune pathways and the RNA interference-antiviral signaling pathway, have been identified as playing a role in pathogen transmission [3, 63]. However, in our comparisons using VectorBase, we did not find any correlation between the loci we identified and these specific pathways.

## Conclusions

This comprehensive study provides the first in-depth analysis of genetic diversity in *H. marginatum* in Türkiye, revealing significant genetic differentiation among populations, particularly in CCHF-endemic regions. While higher genetic variation in harsh environments where the CCHF virus (CCHFV) is prevalent could enhance fitness, survival, and adaptation in tick species [64], it remains unclear whether the virus itself exerts selective pressure on tick survival or development, as observed in other tick-borne infections such as tick-borne encephalitis or *Borrelia* [65, 66]. For instance, *Anaplasma phagocytophilum* infection has been shown to increase heat shock proteins (HSPs) in tick cells, improving tick survival and questing behavior, thus facilitating pathogen transmission and adaptation to harsh conditions [67, 68]. However, there is also no substantial evidence suggesting significant detrimental impacts of CCHFV on tick survival or development, as studies indicate that CCHFV has adapted to its tick hosts, pointing to a long co-evolutionary relationship [64]. Therefore, although our study captures a relationship between higher genetic diversity and CCHFV incidence, this does not necessarily indicate adaptation to CCHFV, as persistent genetic diversity may also be shaped by environmental factors, host community structures, or the tick microbiome rather than CCHFV infection alone. In regions where CCHF circulates at lower incidence, alternative selective forces such as environmental pressures or host interactions may play a more prominent role. These findings underscore the need for genome-wide studies that integrate factors such as host movements, hybridization, environmental conditions, and socioeconomic influences on human exposure to infectious bites [69]. Furthermore, considering the recently identified role of the tick microbiome in influencing vectorial capacity [70], a multifaceted approach is essential for understanding the drivers of genetic variation and their implications for vector-borne disease transmission and control strategies.

## Supplementary Information

Supplementary Material 1: Fig. 1. Genome-wide nucleotide diversity (èð) across seven chromosomes of *H. marginatum* populations in Türkiye. The *y*-axis represents nucleotide diversity (èð) calculated in 50-kb sliding windows with a 25-kb step size, while the *x*-axis indicates chromosomal positions in base pairs. Each plot corresponds to a distinct chromosome (GWHCBGJ00000001–GWHCBGJ00000007), displaying the variation in diversity across genomic regions.

Supplementary Material 2: Fig. 2. 31 SNPs that are significantly associated with genetic differentiation

Supplementary Material 3.

## Data Availability

Raw sequence reads will be deposited at NCBI Sequence Read Archive (SRA).
