# Supplementary figures and images for: High Crimean-Congo hemorrhagic fever incidence linked to greater genetic diversity and differentiation in Hyalomma marginatum populations in Türkiye
Source: Parasit Vectors. 2024 Nov 19;17:477. doi: 10.1186/s13071-024-06530-z (PMC11590318; doi:10.1186/s13071-024-06530-z)

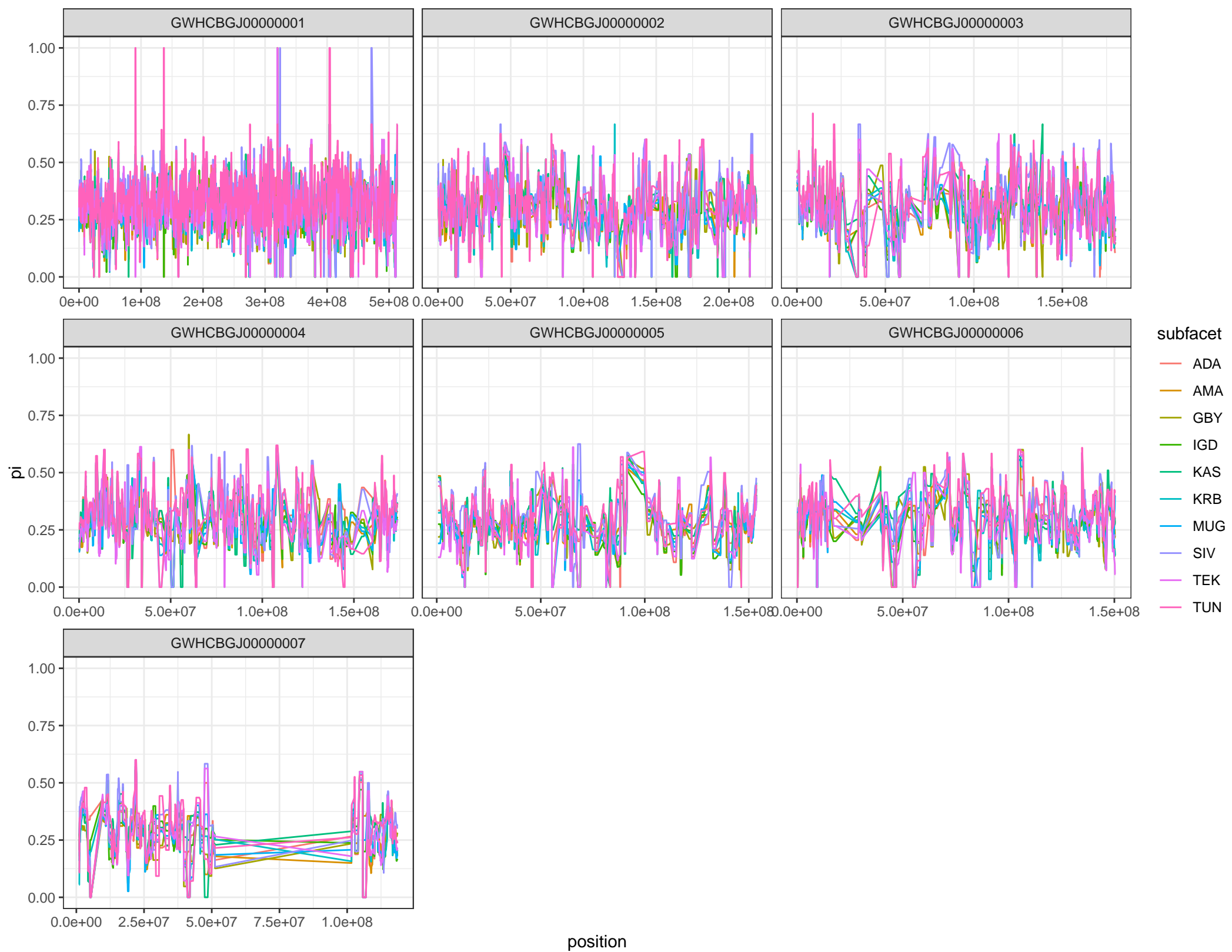

Supplement: Supplementary file 1 — Supplementary Material 1: Fig. 1. Genome-wide nucleotide diversity (èð) across seven chromosomes of H. marginatum populations in Türkiye. The y-axis represents nucleotide diversity (èð) calculated in 50-kb sliding windows with a 25-kb step size, while the x-axis indicates chromosomal positions in base pairs. Each plot corresponds to a distinct chromosome (GWHCBGJ00000001–GWHCBGJ00000007), displaying the variation in diversity across genomic regions. [file 13071_2024_6530_MOESM1_ESM.pdf]

# Loading Plot for DA 1

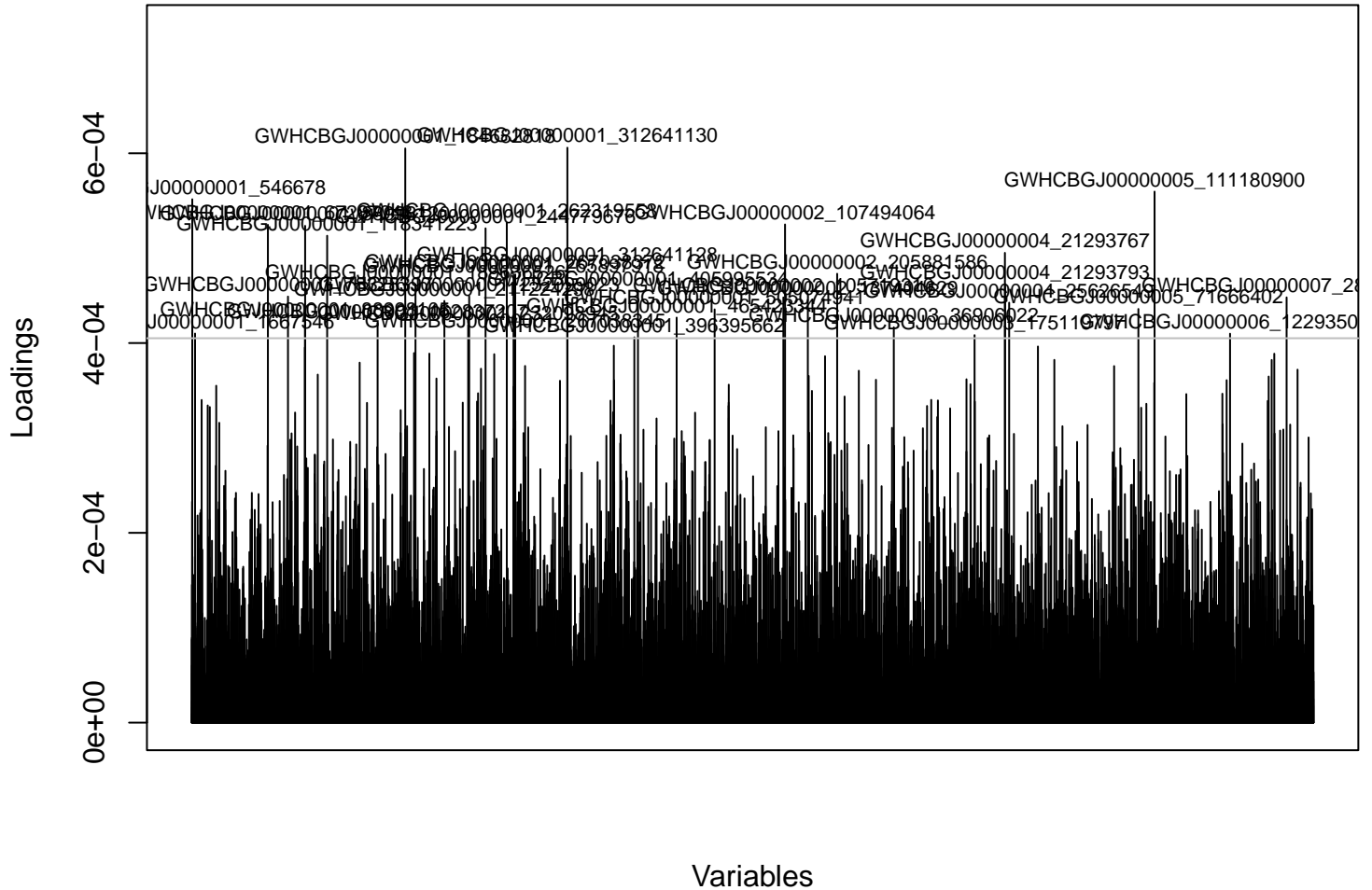

Supplement: Supplementary file 2 — Supplementary Material 2: Fig. 2. 31 SNPs that are significantly associated with genetic differentiation [file 13071_2024_6530_MOESM2_ESM.pdf]
