# Supplementary material for: High Crimean-Congo hemorrhagic fever incidence linked to greater genetic diversity and differentiation in Hyalomma marginatum populations in Türkiye
Source: Parasit Vectors. 2024 Nov 19;17:477. doi: 10.1186/s13071-024-06530-z (PMC11590318; doi:10.1186/s13071-024-06530-z)
Supplement: Supplementary file 3 — Supplementary Material 3. [file 13071_2024_6530_MOESM3_ESM.docx]

**Supplementary Tables:**

Supplementary Table 1. Geographic coordinates of sampling localities, and tick collection methods

| **Code** | **Province** | **Latitude** | **Longitude** | **Method(Host)** |
| --- | --- | --- | --- | --- |
| ADA | Adapazarı | 40.77763 | 30.62295 | cow |
| AMA | Amasya | 40.789 | 35.677 | cow |
| GUM | Gümüşhane | 40.030 | 39.514 | cow |
| KAS | Kastamonu | 41.611047 | 33.116154 | cow |
| KRB | Karabük | 40.874766 | 32.644286 | cow |
| IGD | Iğdır | 39.821 | 43.813 | cow |
| MUG | Muğla | 37.06589 | 27.24833 | cow |
| SIV | Sivas | 39.144875 | 37.372032 | cow |
| TEK | Tekirdağ | 41.22361 | 27.248333 | cow |
| TUN | Tunceli | 39.46027 | 40.02111 | cow |

Supplementary Table 2. Population information and raw sequence and alignment numbers of the samples (Samples used in this study were marked as yellow)

| Population | Individual | Total_Reads | Mapped_reads | Paired&Mated | Properly_Aligned | Percent_Aligned |
| --- | --- | --- | --- | --- | --- | --- |
| Adapazarı | ADA101 | 4618178 | 2699522 | 2637261 | 2090664 | 58 |
| Adapazarı | ADA102 | 9860172 | 8798154 | 8579597 | 6285514 | 89 |
| Adapazarı | ADA103 | 16384807 | 14908115 | 14533774 | 10507888 | 90 |
| Adapazarı | ADA104 | 18118701 | 16226129 | 15831317 | 11550492 | 89 |
| Adapazarı | ADA106 | 18474270 | 14951817 | 14574693 | 10944906 | 80 |
| Adapazarı | ADA10 | 7295163 | 3445539 | 2866903 | 2339677 | 47 |
| Adapazarı | ADA11 | 6028574 | 3049105 | 2633809 | 2220644 | 50 |
| Adapazarı | ADA1 | 5453869 | 4991742 | 4883988 | 3642578 | 91 |
| Adapazarı | ADA4 | 7163425 | 1099037 | 1044403 | 1020478 | 15 |
| Adapazarı | ADA5 | 9884721 | 9143867 | 8941726 | 6644074 | 92 |
| Adapazarı | ADA6 | 4642421 | 4090816 | 3992013 | 2928274 | 88 |
| Adapazarı | ADA8 | 16452736 | 15388180 | 15029767 | 10988568 | 93 |
| Adapazarı | ADA9 | 19591258 | 18346472 | 17842671 | 12762386 | 93 |
| Amasya | AMA101 | 15238571 | 14417190 | 14093993 | 10433752 | 94 |
| Amasya | AMA102 | 14624313 | 13821333 | 13516663 | 9999479 | 94 |
| Amasya | AMA105 | 9517598 | 8957706 | 8745082 | 6461906 | 94 |
| Amasya | AMA106 | 14911475 | 14041167 | 13745898 | 10377317 | 94 |
| Amasya | AMA4 | 10163209 | 5367767 | 5202542 | 4160339 | 52 |
| Amasya | AMA6 | 6393530 | 6071043 | 5932019 | 4346800 | 94 |
| Amasya | AMA8 | 19354887 | 18242437 | 17794055 | 12894819 | 94 |
| Amasya | AMA9 | 13468701 | 12760293 | 12481602 | 9193512 | 94 |
| Gümüşhane | BAY18 | 876735 | 834281 | 818278 | 576250 | 95 |
| Gümüşhane | BAY22 | 2036351 | 1908534 | 1880144 | 1431360 | 93 |
| Gümüşhane | BAY26 | 2240868 | 1978192 | 1935482 | 1444395 | 88 |
| Gümüşhane | BAY4 | 1454705 | 1293569 | 1272397 | 982122 | 88 |
| Gümüşhane | BAY6 | 5781 | 2707 | 2629 | 1449984 | 46 |
| Gümüşhane | GUM100 | 2610150 | 2267925 | 2239009 |  | 86 |
| Gümüşhane | GUM101 | 727185 | 575922 | 567309 | 355269 | 79 |
| Gümüşhane | GUM102 | 1476776 | 1159316 | 1139435 | 850807 | 78 |
| Gümüşhane | GUM103 | 1463841 | 1332918 | 1311732 | 935469 | 91 |
| Gümüşhane | GUM23 | 2347176 | 2226008 | 2185543 | 1668333 | 94 |
| Iğdır | IGD12 | 18023755 | 17085088 | 16703650 | 12356899 | 94 |
| Iğdır | IGD18 | 15363441 | 14417623 | 14092585 | 10424829 | 93 |
| Iğdır | IGD20 | 14193634 | 13444941 | 13148101 | 9792105 | 94 |
| Iğdır | IGD22 | 8660973 | 8117891 | 7940531 | 5933824 | 93 |
| Iğdır | IGD23 | 14738969 | 13844356 | 13514969 | 9806631 | 93 |
| Iğdır | IGD25 | 13644675 | 12819931 | 12525197 | 9247917 | 93 |
| Iğdır | IGD26 | 8519817 | 8031581 | 7845828 | 5851129 | 94 |
| Iğdır | IGD27 | 17181862 | 16194094 | 15820513 | 11706834 | 94 |
| Iğdır | IGD29 | 9778913 | 9138657 | 8911788 | 6431031 | 93 |
| Iğdır | IGD31 | 8911428 | 8198321 | 8008813 | 6035727 | 91 |
| Iğdır | IGD32 | 7375270 | 7171238 | 7105711 | 6230929 | 97 |
| Iğdır | IGD7 | 7060183 | 6679542 | 6526021 | 4776625 | 94 |
| Kastamonu | KAS17-11 | 1958993 | 529555 | 512363 | 450532 | 27 |
| Kastamonu | KAS17-12 | 6813039 | 6142518 | 6005951 | 4449676 | 90 |
| Kastamonu | KAS17-13 | 6670621 | 5865795 | 5737792 | 4295841 | 87 |
| Kastamonu | KAS17-14 | 4128001 | 1164386 | 1126840 | 990005 | 28 |
| Kastamonu | KAS17-15 | 4718391 | 3355827 | 3283147 | 2478583 | 71 |
| Kastamonu | KAS17-16 | 3012566 | 1147952 | 1116853 | 911621 | 38 |
| Kastamonu | KAS17-17 | 12117591 | 10252976 | 9978366 | 7299005 | 84 |
| Kastamonu | KAS17-18 | 18156176 | 10467756 | 10148736 | 7743697 | 57 |
| Karabük | KRB17-1 | 19783703 | 18666307 | 18210072 | 13322697 | 94 |
| Karabük | KRB17-2 | 13364612 | 12666928 | 12399774 | 9197260 | 94 |
| Karabük | KRB17-3 | 19702031 | 18543244 | 18104047 | 13235588 | 94 |
| Karabük | KRB17-4 | 7919344 | 7440427 | 7288049 | 5466945 | 93 |
| Karabük | KRB17-5 | 13882271 | 12976648 | 12648951 | 9202984 | 93 |
| Karabük | KRB17-6 | 23642788 | 22403665 | 21926381 | 16388901 | 94 |
| Karabük | KRB17-7 | 12204244 | 10549441 | 10316616 | 7921998 | 86 |
| Muğla | MUG100 | 13927404 | 13214380 | 12937896 | 9747137 | 94 |
| Muğla | MUG101 | 14284338 | 13574659 | 13300667 | 10017245 | 95 |
| Muğla | MUG102 | 282042 | 250169 | 246639 | 198067 | 88 |
| Muğla | MUG104 | 10284238 | 9622381 | 9396340 | 7016716 | 93 |
| Muğla | MUG105 | 5252219 | 5018271 | 4933452 | 3784601 | 95 |
| Muğla | MUG107 | 12852848 | 12155801 | 11903538 | 8981416 | 94 |
| Muğla | MUG108 | 11383442 | 10783066 | 10535444 | 7813873 | 94 |
| Muğla | MUG109 | 5503188 | 5222740 | 5118817 | 3884453 | 94 |
| Muğla | MUG110 | 12987913 | 12274696 | 12003792 | 8973726 | 94 |
| Muğla | MUG111 | 6156389 | 5834419 | 5707498 | 4301499 | 94 |
| Sivas | SIV10 | 1244712 | 464927 | 452614 | 379010 | 37 |
| Sivas | SIV11 | 1737677 | 1048345 | 1026504 | 813924 | 60 |
| Sivas | SIV13 | 381299 | 71706 | 69279 | 68140 | 18 |
| Sivas | SIV15 | 5051288 | 3837681 | 3749842 | 2861605 | 75 |
| Sivas | SIV17 | 4060893 | 3540206 | 3469857 | 2654213 | 87 |
| Sivas | SIV18 | 1243199 | 1079420 | 1055272 | 799727 | 86 |
| Sivas | SIV3 | 947679 | 201206 | 195784 | 191109 | 21 |
| Sivas | SIV4 | 5489630 | 5101839 | 5000222 | 3795594 | 92 |
| Sivas | SIV6 | 50540 | 26640 | 26299 | 23410 | 52 |
| Sivas | SIV9 | 1560743 | 1086580 | 1062693 | 822950 | 69 |
| Tekirdağ | TEK1-1 | 9484 | 8816 | 8620 | 6295 | 92 |
| Tekirdağ | TEK1-2 | 14221 | 12698 | 12403 | 8065 | 89 |
| Tekirdağ | TEK1-3 | 8862 | 8113 | 7913 | 5259 | 91 |
| Tekirdağ | TEK15 | 2922170 | 1456585 | 1424003 | 1151213 | 49 |
| Tekirdağ | TEK16 | 5738 | 5215 | 5130 | 3680 | 90 |
| Tekirdağ | TEK18 | 19481062 | 18182836 | 17754642 | 12983686 | 93 |
| Tekirdağ | TEK3-1 | 21561826 | 20248056 | 19779898 | 14427840 | 93 |
| Tekirdağ | TEK3-2 | 22153472 | 20556992 | 20102810 | 14775989 | 92 |
| Tekirdağ | TEK9-1 | 7305 | 6667 | 6568 | 4683 | 91 |
| Tekirdağ | TEK9-2 | 10612 | 9718 | 9497 | 6482 | 91 |
| Tunceli | TUN11 | 6619242 | 6286745 | 6147996 | 4522177 | 94 |
| Tunceli | TUN12 | 4878546 | 4616900 | 4513825 | 3324074 | 94 |
| Tunceli | TUN13 | 20692689 | 4466975 | 4182568 | 3897807 | 21 |
| Tunceli | TUN14 | 7239068 | 5036543 | 4894364 | 3662339 | 69 |
| Tunceli | TUN5 | 5485 | 4993 | 4899 | 3252 | 91 |
| Tunceli | TUN7 | 3827 | 3466 | 3408 | 2123 | 90 |

Supplementary Table 3. Theta Pi

| **Population** | **mean** | **sd** | **median** | **min** | **max** |
| --- | --- | --- | --- | --- | --- |
| GUM | 0.033 | 0.024 | 0.027 | 0.000 | 0.163 |
| SIV | 0.027 | 0.023 | 0.022 | 0.000 | 0.157 |
| TUN | 0.026 | 0.022 | 0.021 | 0.000 | 0.179 |
| AMA | 0.019 | 0.017 | 0.015 | 0.000 | 0.124 |
| KAS | 0.025 | 0.021 | 0.020 | 0.000 | 0.170 |
| KRB | 0.018 | 0.016 | 0.014 | 0.000 | 0.136 |
| IGD | 0.018 | 0.017 | 0.014 | 0.000 | 0.133 |
| ADA | 0.020 | 0.018 | 0.016 | 0.000 | 0.137 |
| MUG | 0.020 | 0.018 | 0.015 | 0.000 | 0.139 |
| TEK | 0.018 | 0.016 | 0.014 | 0.000 | 0.137 |

Supplementary Table 4. Tajima’s D

| **Population** | **mean** | **sd** | **median** | **min** | **max** |
| --- | --- | --- | --- | --- | --- |
| GUM | -0.540 | 0.747 | -0.649 | -1.927 | 1.971 |
| SIV | 0.187 | 0.815 | 0.113 | -1.475 | 2.228 |
| TUN | 0.327 | 0.833 | 0.285 | -1.411 | 2.193 |
| AMA | 0.677 | 0.961 | 0.723 | -1.965 | 2.642 |
| KAS | 0.188 | 0.870 | 0.112 | -1.753 | 2.351 |
| KRB | 0.712 | 0.959 | 0.754 | -1.853 | 2.601 |
| IGD | 0.734 | 0.930 | 0.800 | -2.017 | 2.604 |
| ADA | 0.496 | 0.943 | 0.505 | -1.844 | 2.548 |
| MUG | 0.632 | 0.946 | 0.654 | -1.857 | 2.636 |
| TEK | 0.890 | 0.828 | 1.027 | -1.393 | 2.320 |
